# Gamma irradiation-engineered macrophage-derived exosomes as potential immunomodulatory therapeutic agents

**DOI:** 10.1371/journal.pone.0303434

**Published:** 2024-06-12

**Authors:** Hanui Lee, Seong Hee Kang, Gyeong Han Jeong, Seoung Sik Lee, Byung Yeoup Chung, Geun-Joong Kim, Hyoung-Woo Bai

**Affiliations:** 1 Radiation Biotechnology Division, Advanced Radiation Technology Institute (ARTI), Korea Atomic Energy Research Institute (KAERI), Jeongeup, Republic of Korea; 2 Department of Biological Sciences and Research Center of Ecomimetics, College of Natural Science, Chonnam National University, Gwangju, Republic of Korea; 3 Radiation Biotechnology and Applied Radioisotope Science, University of Science and Technology (UST), Daejeon, Republic of Korea; Martin Luther University, GERMANY

## Abstract

The modulation of macrophage polarization is a promising strategy for maintaining homeostasis and improving innate and adaptive immunity. Low-dose ionizing radiation has been implicated in macrophage immunomodulatory responses. However, studies on the relationship between exosomes and regulation of macrophage polarization induced by ionizing radiation are limited. Therefore, this study investigated the alterations in macrophages and exosomes induced by gamma irradiation and elucidated the underlying mechanisms. We used the mouse macrophage cell line RAW 264.7 to generate macrophages and performed western blot, quantitative reverse transcription-PCR, and gene ontology analyses to elucidate the molecular profiles of macrophage-derived exosomes under varying treatment conditions, including 10 Gy gamma irradiation. Exosomes isolated from gamma-irradiated M1 macrophages exhibited an enhanced M1 phenotype. Irradiation induced the activation of NF-κB and NLRP3 signaling in M1 macrophages, thereby promoting the expression of pro-inflammatory cytokines. Cytokine expression was also upregulated in gamma-irradiated M1 macrophage-released exosomes. Therefore, gamma irradiation has a remarkable effect on the immunomodulatory mechanisms and cytokine profiles of gamma-irradiated M1 macrophage-derived exosomes, and represents a potential immunotherapeutic modality.

## Introduction

Immune cells play key roles in homeostasis, wound healing, and immune response [1–3]. Macrophages are crucial immune regulatory cells that recognize pathogens or antigens and produce type I interferons (IFNs) to induce the expression of effector molecules, such as cytokines and chemokines [4, 5]. Macrophages are derived through differentiation of bone marrow monocytes—a subset of white blood cells that occur in systemic circulation and differentiate into macrophages and dendritic cells. Circulating monocytes migrate to most tissues in the body where they differentiate into functionally distinct mature macrophages [6, 7]. Macrophages are commonly activated in various microenvironmental signaling pathways and differentiate into the M1 (antitumor and pro-inflammatory) and M2 subtypes (protumor and anti-inflammatory) [8]. M1 macrophages are polarized by lipopolysaccharide (LPS) and IFN-γ stimulation, resulting in their activation, generation of various pro-inflammatory cytokines such as interleukin (IL)-1β and IL-6, and removal of pathogen and tumor cells. In contrast, M2 macrophages are stimulated by cytokines such as IL-4 and IL-13, leading to tumor growth factor (TGF)-β and IL-12 production, and promoting tissue regeneration and tumor growth [9, 10].

Exosomes, small vesicles enclosed by lipid bilayer membranes that are secreted by all living cells, potentially participate in cell-to-cell interactions. Exosomes exhibit excellent stability, biocompatibility, low immunogenicity, and low toxicity [11, 12]. Importantly, exosomes can facilitate macrophage polarization. For example, M1 macrophage-derived exosomes can reprogram M2 macrophages into M1 macrophages [13]. Given their potential antitumor roles, the biochemical and physicochemical features of exosomes have been extensively investigated.

Low-dose ionizing radiation has emerged as a promising technique in exosome engineering. Ionizing radiation triggers cellular reprogramming by eliciting or inhibiting specific stress signaling pathways, thereby altering the profiles of secreted exosomes [14, 15]. Both the protein and miRNA profiles of blood mononuclear cell-derived exosomes were affected by 2 Gy gamma radiation, suggesting variation in the roles of exosomes in cell–cell communication and microenvironmental regulation [16]. Radiation treatment at 8 Gy dramatically increased the cellular uptake of mesenchymal stem cell-derived exosomes through CD29/CD81 complex formation [17]. However, few studies have investigated the effects of radiation on M1-polarized macrophages and macrophage-derived exosomes.

This study aimed to establish gamma-irradiated M1 macrophage-derived exosomes (IR-M1 exosomes) and determine their potential functions in vitro.

## Materials and methods

### Cell culture

The mouse macrophage cell line RAW 264.7 was purchased from the Korean Cell Line Bank (Seoul, Korea) and cultured in Dulbecco’s modified Eagle’s medium (DMEM; Gibco, Foster City, CA, USA) or Roswell Park Memorial Institute (RPMI) medium (Gibco) supplemented with 10% fetal bovine serum (FBS, Gibco) and 1% (v/v) penicillin–streptomycin (Gibco) at 37°C and 5% CO_2_.

### Preparation of M1 and IR-M1

To induce M1 macrophages, RAW 264.7 cells were treated with 100 ng/mL LPS and 20 ng/mL IFN-γ for 24 h. To prepare IR-M1 macrophages, we first determined the non-cytotoxic radiation doses. We exposed cells to 2–20 Gy and found that 2–10 Gy had no significant impact on cellular toxicity, thus 10 Gy was selected for subsequent investigations (S1 Fig). M1 macrophages were irradiated (10 Gy) at the Advanced Radiation Technology Institute, Korea Atomic Energy Research Institute (Jeongup, Korea) using a Gammacell 40 Exactor with a Cs-137 source. After irradiation, IR-M1 macrophages were incubated at 37°C for 12 h and the expression of iNOS and GAPDH was determined by western blot analysis. To inhibit the activation of caspase-1, cells were pretreated with 20 μM caspase-1 inhibitor (Ac-YVAD-CMK, Sigma-Aldrich, St. Louis, MO, USA) for 1 h before LPS treatment.

### Preparation and characterization of MΦ, M1, and IR-M1 exosomes

Exosomes were isolated using a total exosome isolation kit (Invitrogen, Carlsbad, CA, USA) according to the manufacturer’s instructions. Cells were incubated in serum-free media for 12 h, the cell supernatant was collected, and cell debris was eliminated by centrifugation at 2000 × *g* for 30 min. The supernatant was mixed with the exosome isolation reagent (0.5 mL). Following overnight incubation at 4°C, the exosomes were collected by centrifugation at 10, 000 × *g* and 4°C for 1 h. After negative staining with uranium acetate, the morphology of MΦ, M1, and IR-M1 exosomes was evaluated using a transmission electron microscope (Hitachi, Japan). The exosomes were quantified using a bicinchoninic acid (BCA) assay and 0.1 μg/mL total protein was collected for further characterization. The size distribution and concentration of exosomes were investigated using dynamic light scattering (DLS) and nanoparticle tracking analysis (NTA). The expression of exosome biomarkers (ALIX and GAPDH) was verified by western blotting. M1 macrophages were treated with 1 μM doxorubicin for 3 h to prepare Dox-M1 macrophages; Dox-M1 exosomes were prepared using the exosome isolation kit.

### Quantitative reverse-transcription PCR

Total RNA was extracted from MΦ, M1, and IR-M1 macrophages and exosomes using Total RNA isolation reagent (Welgene, Seoul, Korea) according to the manufacturer’s instructions. Exosomal cDNA was prepared from 2 μg of extracted RNA using an M-MLV Reverse Transcriptase cDNA Synthesis Kit (Invitrogen) according to the manufacturer’s instructions. SYBR Green-based qPCR was performed using mouse cytokine-specific primers. mRNA levels were normalized to that of GAPDH (ΔCt = Ct gene of interest − Ct GAPDH) and reported as relative mRNA expression [ΔΔCt  =  2 −(ΔCt sample − ΔCt control)] or fold change values.

### Western blotting

Cells and exosomes were lysed in RIPA buffer, and total protein was collected by centrifugation at 4°C and 13 000 × *g* for 40 min and quantified using the BCA assay [18]. Next, 10 μg of the protein was subjected to sodium dodecyl sulfate-polyacrylamide gel electrophoresis, transferred onto polyvinylidene difluoride membranes blocked with non-fat milk, and incubated overnight at 4°C with primary antibodies against iNOS, ALIX, IκB-a, NF-κB, COX-2, and GAPDH (1:1,000, Cell Signaling Technology, Beverly, MA, USA). The membranes were then washed and incubated with a secondary anti-rabbit antibody for 2 h at room temperature. Finally, the intensity of the protein bands was determined using an ECL kit (Cytiva, Marlborough, MA, USA) according to the manufacturer’s instructions. Membranes were incubated with ECL detection reagent for 1 min at room temperature. After removing the solution, blots were visualized with an iBrightCL1000 (Invitrogen).

### Statistical analysis

All data were evaluated using Student’s *t*-test with statistical significance set at *p* < 0.05. Experiments were independently performed at least three times.

## Results

### Preparation and characterization of MΦ, M1, and IR-M1 exosomes

**Fig 1:**
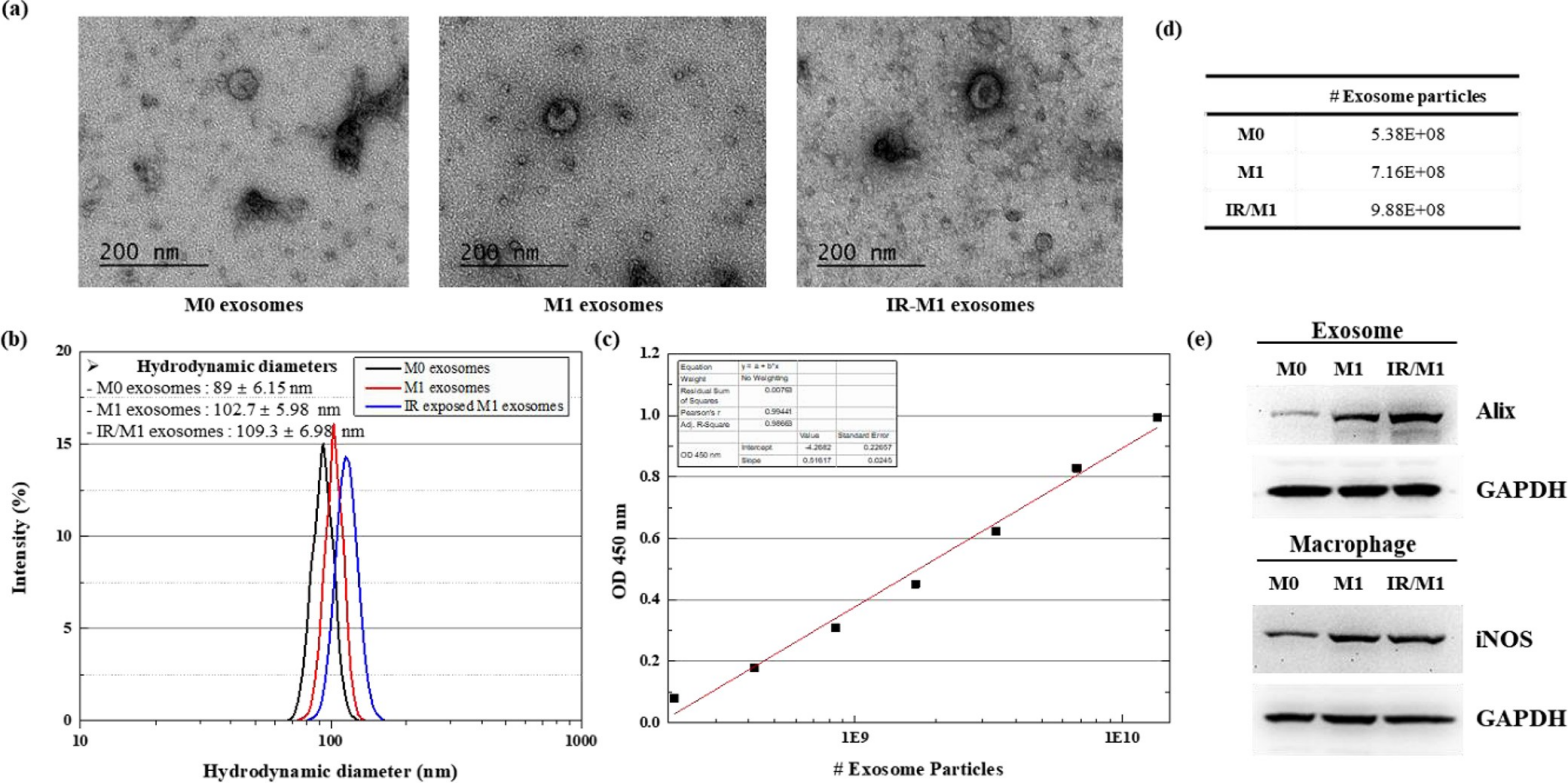
Characterization of MΦ, M1, and gamma-irradiated M1-derived exosomes. (a) Representative TEM image of exosomes following negative staining with uranium acetate. (b) Size distribution of exosomes measured using dynamic light scattering. (c) CD63 protein standard curve. (d) Exosome concentration in MΦ, M1, and radiation exposed M1-macrophage culture medium (e) Expression of M1-macrophage markers iNOS and Alix analyzed by western blotting.

The morphology of MΦ, M1, and IR-M1 exosomes was determined using TEM analysis. TEM images showed that all exosomes were spherical and well dispersed (Fig 1A). The NTA and DLS indicated that the concentration and size of exosomes derived from IR-M1 were greater than those from MΦ and M1 exosomes (Fig 1B and 1C). The western blot analysis showed that ALIX (a general exosomal marker) and iNOS (M1 macrophage marker) were highly expressed in IR-M1 macrophage-derived exosomes (Fig 1E). These results suggested that exosome secretion was stimulated by gamma radiation.

### Exosomes are susceptible to gamma irradiation-induced cytokine expression

**Fig 2:**
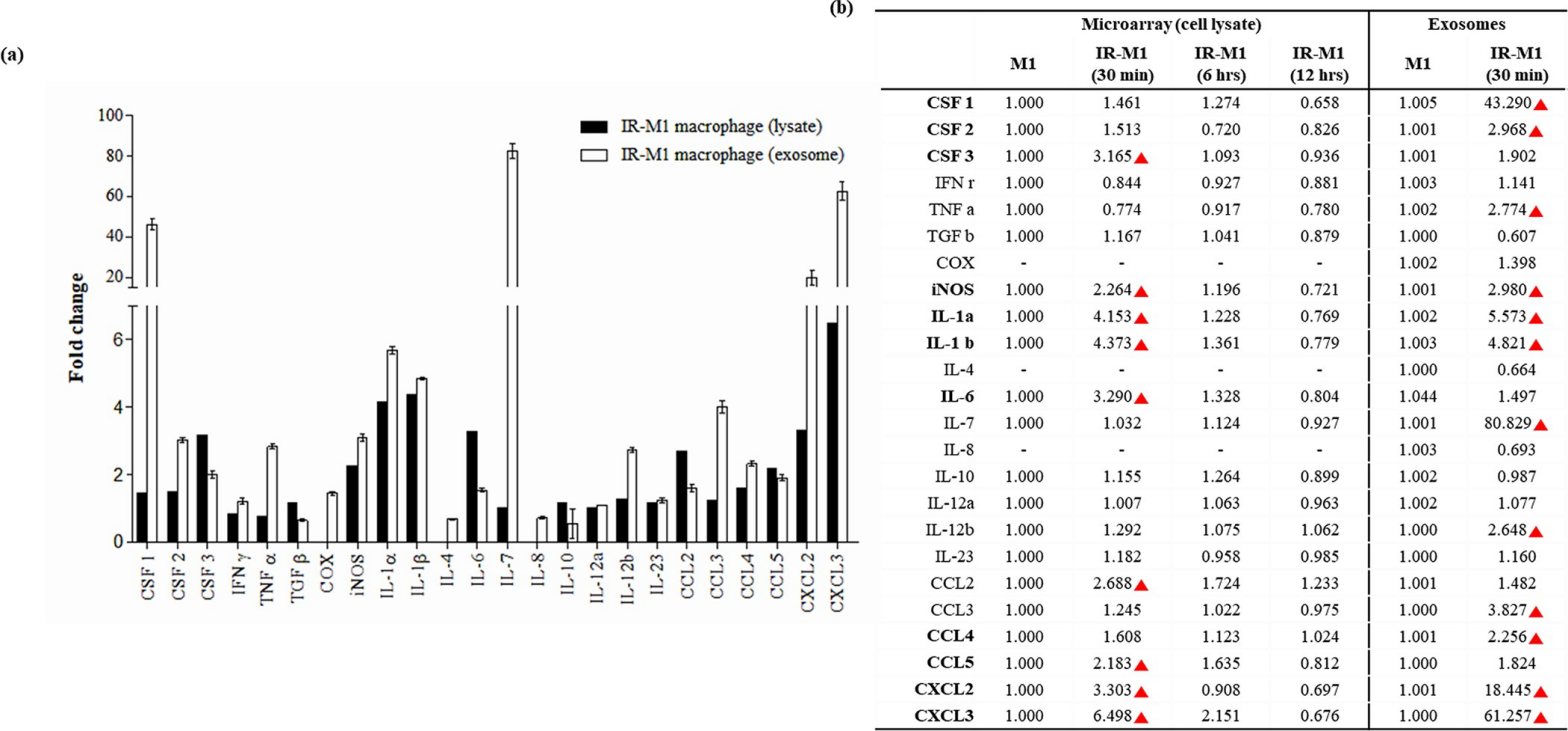
Quantitation of cytokine expression in IR-exposed M1 macrophage or exosomes. (a) Relative mRNA fold changes of pro/anti-inflammatory cytokines and chemokines of cell lysate and exosomes isolated from M1 macrophages 30 minutes after irradiation. (b) Statistical analysis of cytokine array data from M1 cell lysates 0.5, 6, and 12 hours after irradiation compared to exosome data.

**Fig 3:**
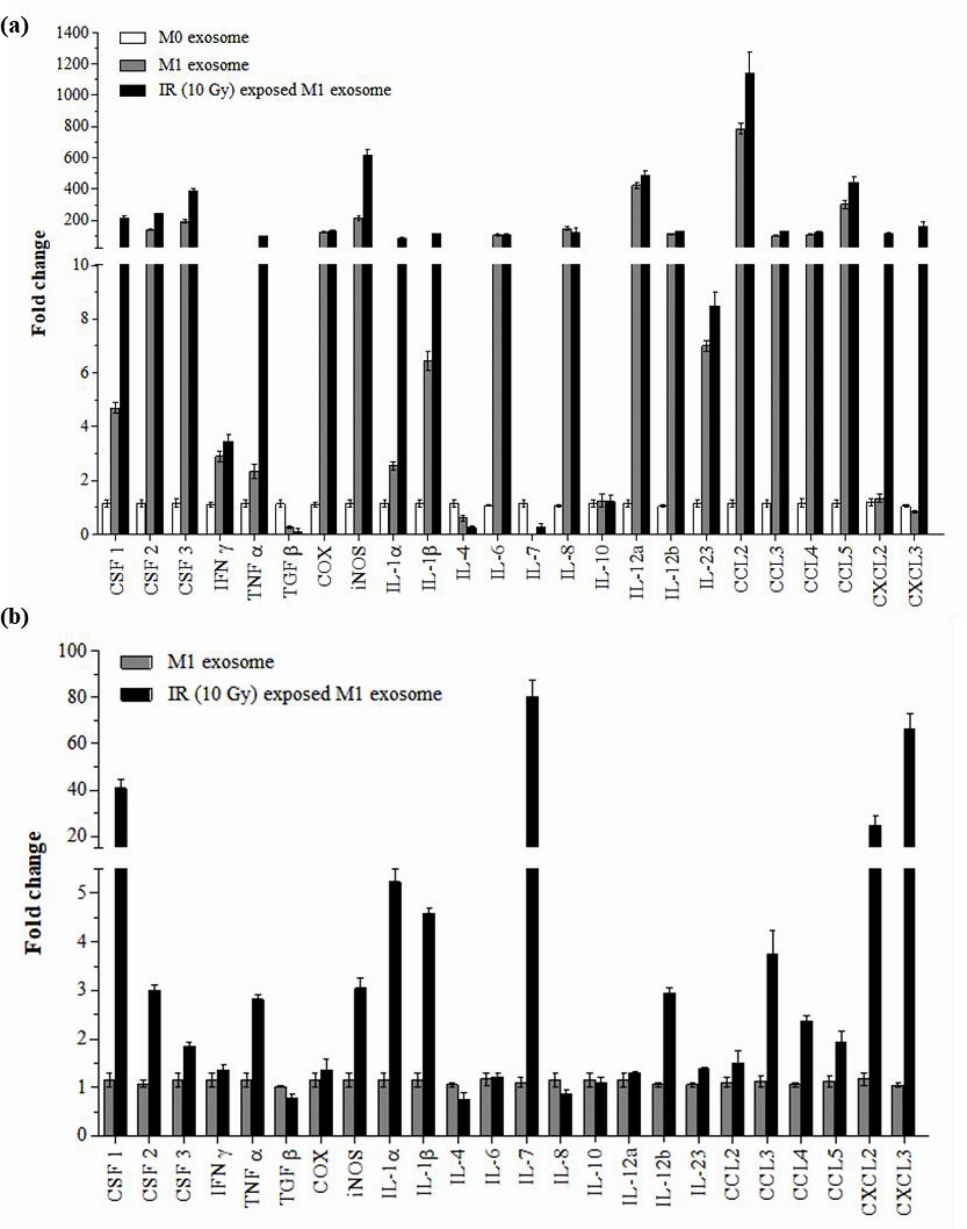
Relative mRNA fold change of pro-/anti-inflammatory cytokines and chemokines of exosomes. (a) Cytokine expression of MΦ, M1, and radiation-exposed M1 exosomes was determined through qPCR. (b) Cytokine expression of irradiated M1 macrophage-derived exosomes was compared with M1 exosomes.

The mRNA levels of pro-inflammatory cytokines and chemokines were compared to elucidate the inflammatory response induced by gamma irradiation. mRNA was isolated at 30 min, 6 h, and 12 h after gamma irradiation, and cytokine levels were determined using a cDNA microarray and mouse cytokine multi-analyte protein array. The microarray data presented in the levels of cytokines in IR-M1 exosomes isolated 30 min after irradiation were significantly upregulated compared with those in cell lysates (Fig 2A), suggesting that IR-M1-derived exosomes may mediate immune regulation in inflammatory diseases. The microarray analysis showed that the expression of the pro-inflammatory cytokines IL-1α, IL-1β, and CXCL3 was significantly higher 0.5 h after radiation exposure (Fig 2B). The levels of CSF3, IL-6, CCL2, CSF1, and IL-7 varied between exosomes and cell lysates, suggesting potential variations in cargo transfer processes between cells and exosomes. The cytokine levels within M0-, M1-, and IR-M1 macrophage-isolated exosomes were analyzed using qRT-PCR to compare their immunomodulatory profiles. We found that the expression of all cytokines, except TGF-β, IL-4, IL-7, and IL-10, was upregulated by at least threefold in M1 and IR-M1 exosomes compared to M0 exosomes (Fig 3A). We compared cytokine expression between M1 and IR-M1 exosomes using qRT-PCR and found that, in IR-M1 exosomes, the expression of TGF-β, IL-4, and IL-8 was slightly downregulated compared to M1 exosomes, while there was no change in IL-10 and IL-12a expression. The remaining cytokines were expressed at higher levels in IR-M1 exosomes (Fig 3B). These results reflect the impact of gamma irradiation on cytokine expression profiles, and indicate activation of the inflammatory response. The significant upregulation of cytokines in IR-M1 exosomes suggests a potential role in immune regulation.

### Gamma irradiation regulates NLRP3 inflammasome activation via NF-κB signaling pathway

**Fig 4:**
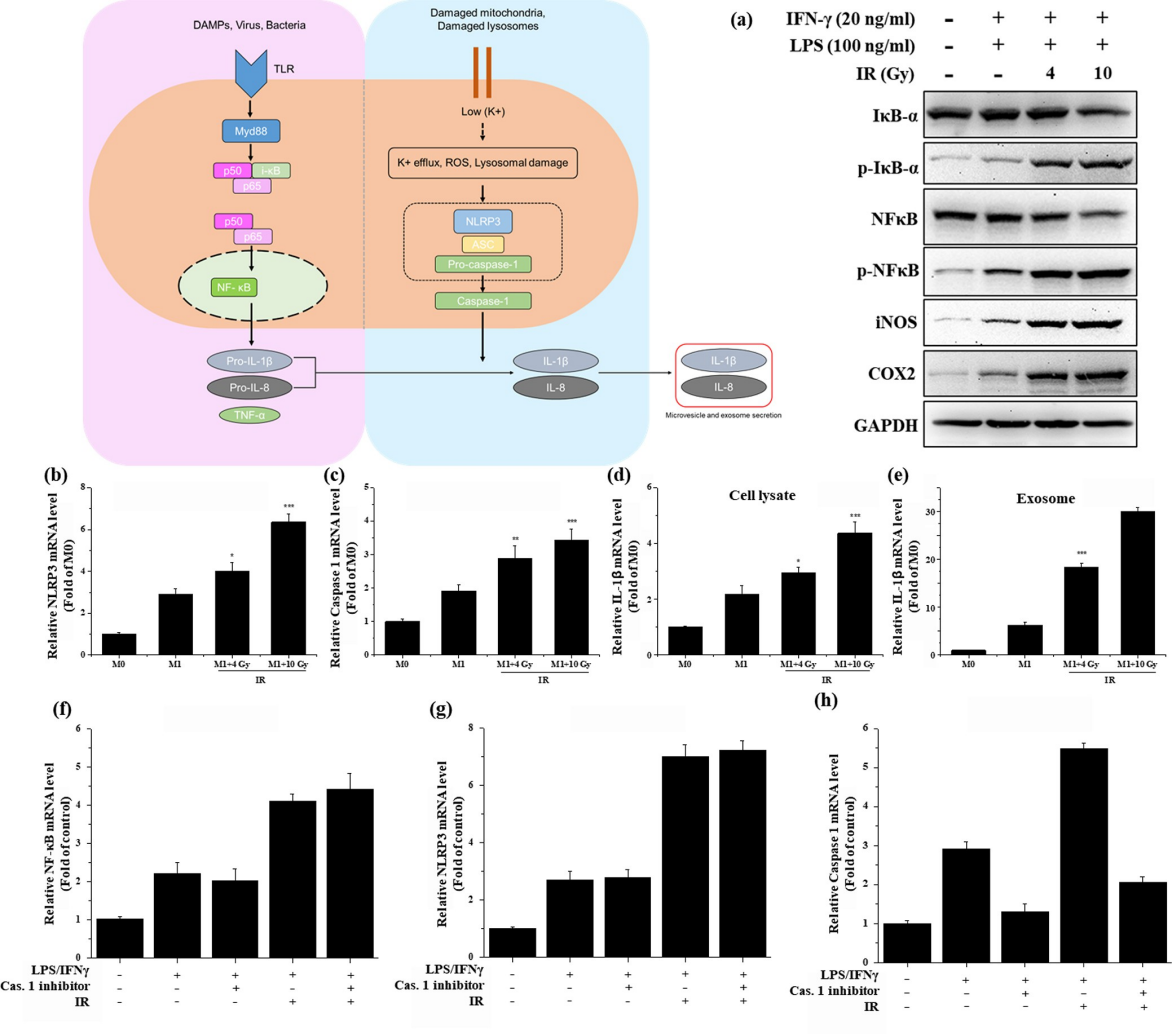
Radiation activates NF-kB and NLRP3 inflammasome signaling pathways in M1 macrophages. (a) Western blot analysis of NF-kB pathway activation. (b) mRNA expression of inflammasome-related genes (NLRP3), detected via qRT PCR. (c) mRNA expression of caspase-1. (d) mRNA expression of IL-1B in cell lysate. (e) Secreted exosomes. (f) Fold change of NF-kB expression. (g) NLRP3 and (h) caspase-1 gene expression in the absence or presence of the caspase-1 inhibitor Ac-YVAD-CMK for 1 h.

Regulation of pro-inflammatory cytokines is mediated by the NF-κB signaling pathway [19]. Since IL-1β is a key mediator of the antitumor immune response, we first investigated the activation of the NF-κB and NLRP3 inflammasome signaling pathways using western blotting (Fig 4A). We found that irradiation increased the phosphorylation levels of IκB-a as well as the expression of iNOS and COX-2 in M1 macrophages. qRT-PCR showed that, as expected, 10 Gy radiation significantly increased NLRP3 and caspase-1 mRNA expression (Fig 4B–4E). IL-1β mRNA levels also increased in cell lysates and exosomes after irradiation. To further clarify the role of ionizing radiation in NF-κB and NLRP3 inflammasome signaling, we treated the cells with the caspase-1 inhibitor and found that the levels of caspase-1 and IL-1β decreased in cell lysates and exosomes (Fig 4F and 4G). However, gamma irradiation effectively reduced caspase-1 and IL-1β levels in a dose-dependent manner. Furthermore, the caspase-1 inhibitor did not cause any significant changes in NF-κB and NLRP3 expression. These observations demonstrate the ability of gamma radiation to elicit cytokine-specific immune responses to activate the NF-κB signaling pathway.

### Increased inflammatory cytokine levels in IR-M1 exosomes

**Fig 5:**
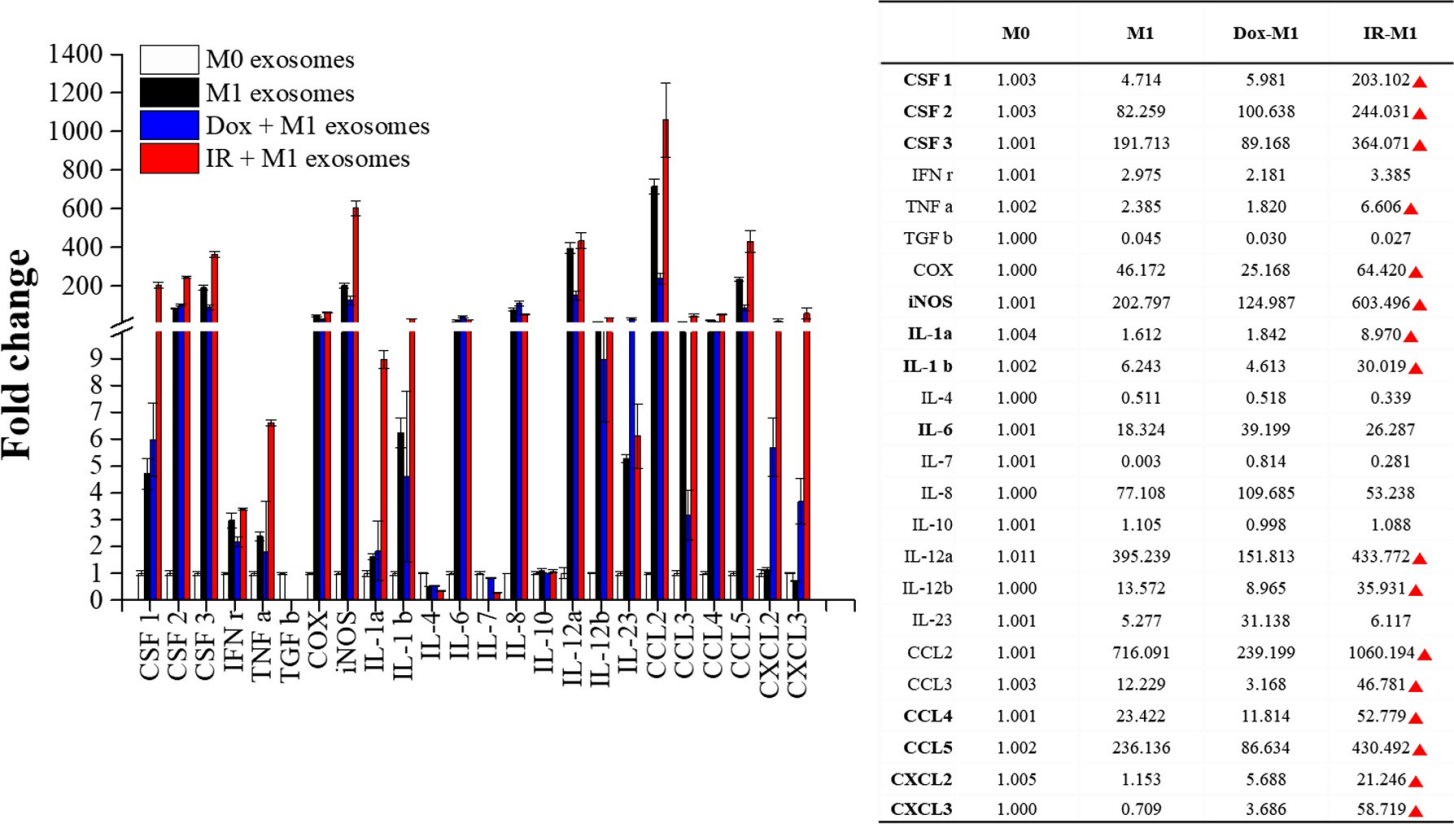
Relative mRNA fold change of pro-/anti-inflammatory cytokines and chemokines in MΦ, M1, Dox-treated M1, and radiation-exposed M1-derived exosomes.

The expression of pro-/anti-inflammatory cytokines and chemokines in MΦ, M1, doxorubicin-treated M1, and radiation-exposed M1-derived exosomes was determined using qRT-PCR (Fig 5). Doxorubicin (an immunomodulatory anticancer agent) was used to treat M1 macrophages as a positive control to validate the immunomodulatory effects of ionizing radiation. Compared to MΦ exosomes, a moderate increase in inflammatory cytokine levels was observed in M1 and Dox-M1 exosomes, except for TGF-β, IL-4, IL-7, and IL-10. However, gamma irradiation of the M1 exosomes significantly increased cytokine expression. These findings reflect the superior immunomodulatory efficacy of gamma radiation, as evidenced by the more effective induction of anti-tumor immune responses by IR-M1 exosomes compared to doxorubicin-treated M1-derived exosomes.

**Fig 6:**
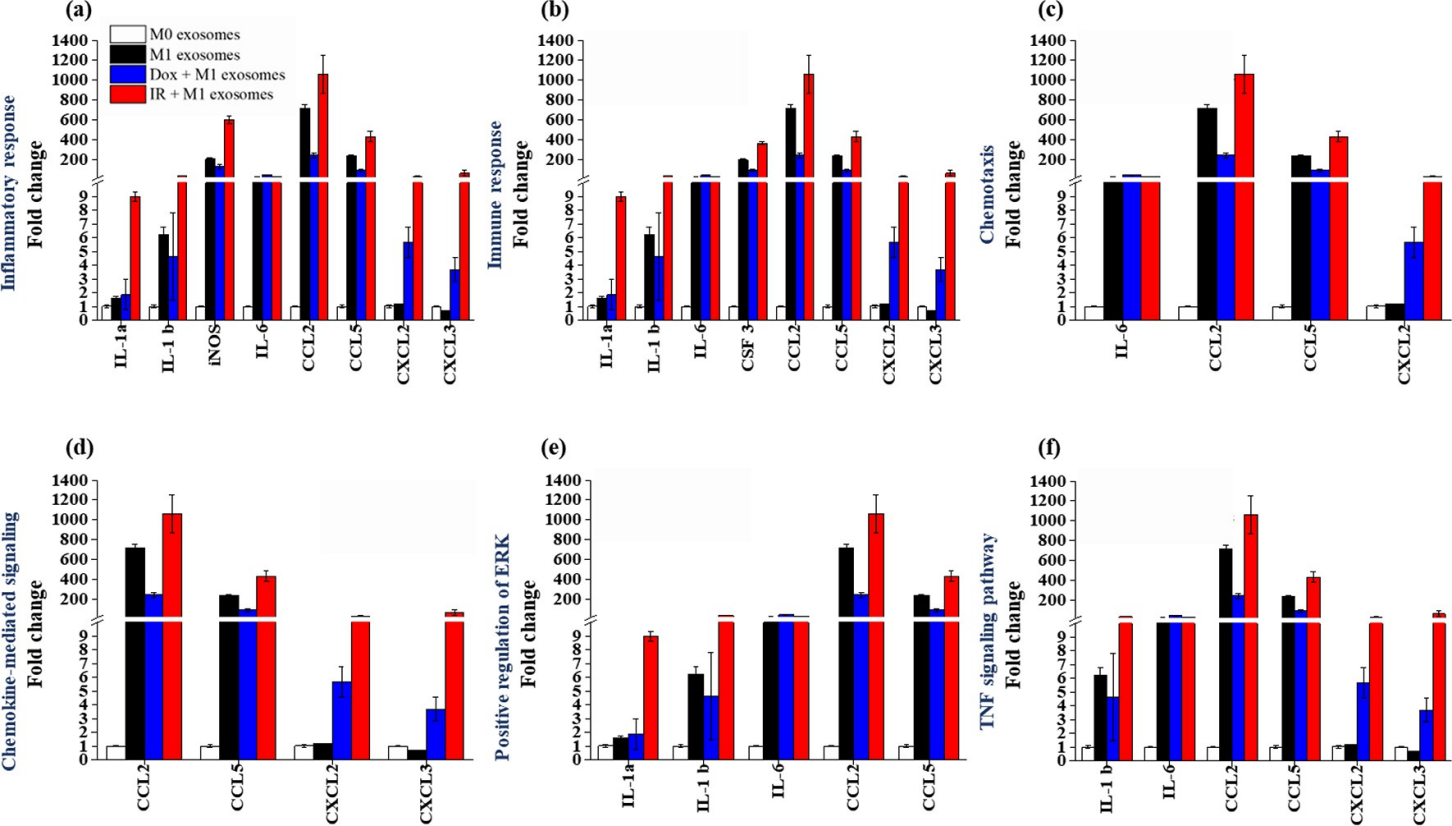
Regulation (RT-qPCR) of various gene ontology categories induced by gamma irradiation in RAW 264.7 macrophage-derived exosomes. (a) Inflammatory response. (b) Immune response. (c) Chemokine-mediated signaling. (d) Positive regulation of ERK. (e) TNF signaling pathway. All data are based on three independent biological replicates.

We further evaluated the immunotherapeutic effects of IR-M1 exosomes based on the differential expression of immune response-related mRNAs (Fig 6). The upregulated cytokine levels in IR-M1 exosomes were associated with the inflammatory response; immune response; chemotaxis; positive regulation of ERK1 and ERK2 cascade; and cellular response to IL-1, IFN-γ, and LPS. In addition, the expression of cytokines associated with TNF-, cytokine-, and chemokine-mediated signaling pathways was highly upregulated in IR-M1 exosomes. These findings highlight the potential immunotherapeutic effects of IR-M1 exosomes, given their distinct profile of differentially expressed mRNAs associated with immune responses.

## Discussion

In this study, we elucidated the effect of gamma irradiation on M1 macrophage-derived exosomes. Gamma irradiation significantly upregulated pro-inflammatory cytokine levels in exosomes via NF-κB and NLRP3 inflammasome pathway activation. We demonstrated, for the first time, that gamma radiation effectively facilitated cytokine expression in M1 macrophage-derived exosomes.

Macrophages play an important protective role in the human body by secreting cytokines and regulating immune responses. The two macrophage subtypes can be induced in response to various stimulating factors in the inflammatory microenvironment. In the early stages of tumor development, M1 macrophages establish a more favorable environment via the increased production of pro-inflammatory cytokines and chemokines. IFN-**γ** and TNF-α can polarize macrophages to the M1 phenotype [20, 21]. The stress response further activates the transcription of genes encoding IL-6 and IL-1β [22]. In contrast, M2 macrophages have anti-inflammatory and wound-healing activities [23]. The M2 phenotype exerts immunosuppressing effects through the actions of IL-10 and TGF-β by activating transcription factors, such as IRF4 [24]. Because M1/M2 ratios are highly associated with disease severity, the regulation of macrophage phenotypes represents a promising treatment approach [25, 26]. Notably, exosomes can alter macrophage phenotypes by regulating cell-cell communication [13, 27].

Considering the importance of exosomes in immune response regulation, various biomedical studies have investigated the roles of exosomes and attempted to enhance their biological activities through molecular engineering. For instance, calcium chloride (CaCl_2_)-mediated transfection can effectively load exosomes with miRNAs [28]. Lee et al. introduced click chemistry to prepare long-lasting fluorescently labeled exosomes as theragnostic agents [29]. However, the effects of radiation treatment on exosomes remained largely unexplored, until now. Our findings provide new insights into the characteristics of exosomes secreted by M1 macrophages following radiation exposure.

Gamma-irradiated M1 macrophage-secreted exosomes showed increased size and marker protein expression compared to MΦ and M1 exosomes. Our findings are consistent with those of previous reports, confirming that exosome cargo and secretion rates are altered after irradiation [30]. Consequently, gamma irradiation is expected to induce the production of pro-inflammatory cytokines. The qRT-PCR analysis demonstrated a significant increase in the expression of inflammatory cytokines in IR-M1 exosomes. Based on their characteristics and the effects of irradiation, exosomes represent promising targets for regulating immune responses in the tumor microenvironment.

We analyzed the protein expression in macrophages following radiation exposure to better demonstrate the role of radiation in immunoregulation. We observed that ionizing radiation (10 Gy) stimulated NF-κB and NLRP3 inflammasome activation, whereas non-irradiated MΦ and M1 macrophages exhibited milder pathway activation. Furthermore, caspase-1 production was blocked by treatment with the caspase-1 inhibitor Ac-YVAD-CMK, which does not inhibit the formation of caspase-1 but rather its activity and biological function, thereby inhibiting NLRP3 inflammasome activities. The caspase-1 inhibitor almost completely eliminated the expression of caspase-1 and IL-1β in macrophages. However, gamma irradiation could restore caspase-1 and its downstream IL-1β expression to normal levels. Therefore, we concluded that gamma irradiation can enhance host immunomodulatory capacity via NF-κB and NLRP3 inflammasome pathway activation.

Although we provide strong evidence for the effects of radiation on M1 macrophage-derived exosome characteristics, a comprehensive analysis of the phenotypic profile is needed to assess the clinical viability of IR-M1 exosomes. Our study has some limitations. First, our experiments only considered murine macrophages and require validation in other cell lines. To fully elucidate the generalizability of the immunomodulating effect of ionizing radiation, further studies using human macrophages should be performed. Long-term studies on macrophage and exosome activity are warranted to maximize the translational potential of ionizing radiation in immunotherapy.

In conclusion, our study provides new insights into the clinical potential of ionizing radiation in immunotherapy. Specifically, gamma irradiation of M1 macrophage-derived exosomes modulated the immune response by altering the cytokine profile toward enhanced therapeutic activity. These findings highlight promising new avenues in immunotherapy.

## Supporting information

S1 Fig Concentration-dependent effects of stimulators on RAW 264.7 macrophages. (a) The Raw264.7 macrophage cells were treated with various dose of gamma ray. Cells were treated with various concentrations of IFN-gamma for 24 h, LPS for 24 h, and doxorubicin for 3 h. After the incubation period, cell viability was determined by MTT assay. (c) The mRNA iNOS level of doxorubicin-treated macrophages were measured using qPCR.
(TIF)

S1 Raw images (PDF)

S2 Raw images (PDF)
