# Supplementary figures and images for: Gamma irradiation-engineered macrophage-derived exosomes as potential immunomodulatory therapeutic agents
Source: PLoS One. 2024 Jun 12;19(6):e0303434. doi: 10.1371/journal.pone.0303434 (PMC11168684; doi:10.1371/journal.pone.0303434)

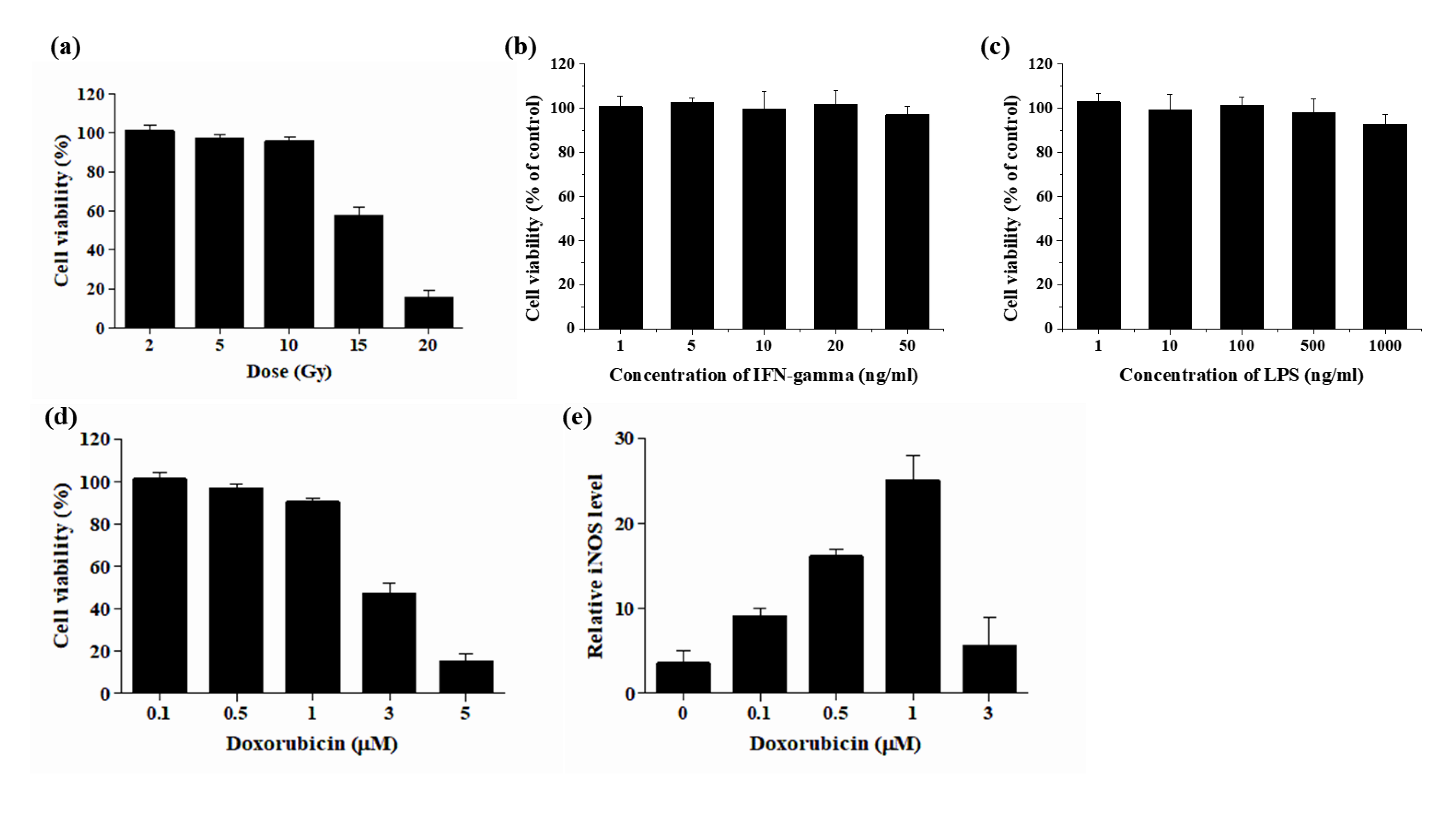

Supplement: S1 Fig — (a) The Raw264.7 macrophage cells were treated with various dose of gamma ray. Cells were treated with various concentrations of IFN-gamma for 24 h, LPS for 24 h, and doxorubicin for 3 h. After the incubation period, cell viability was determined by MTT assay. (c) The mRNA iNOS level of doxorubicin-treated macrophages were measured using qPCR. (TIF) [file pone.0303434.s001.tif]

Full blot images of all western blots reported in Fig 1e:

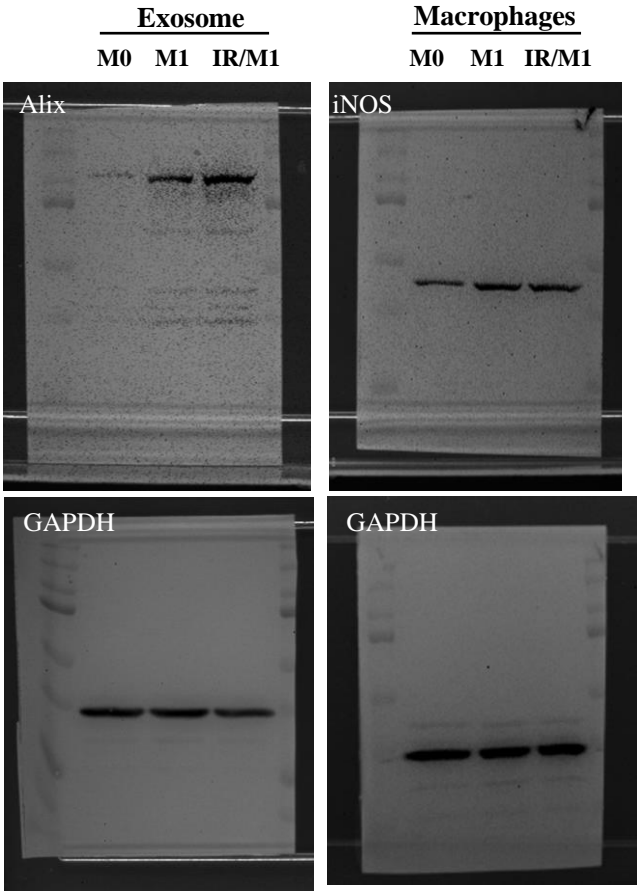

Supplement: S1 Raw images — (PDF) [file pone.0303434.s002.pdf]
