# Supplementary material for: Gamma irradiation-engineered macrophage-derived exosomes as potential immunomodulatory therapeutic agents
Source: PLoS One. 2024 Jun 12;19(6):e0303434. doi: 10.1371/journal.pone.0303434 (PMC11168684; doi:10.1371/journal.pone.0303434)

Full blot images of all western blots reported in Fig 4a

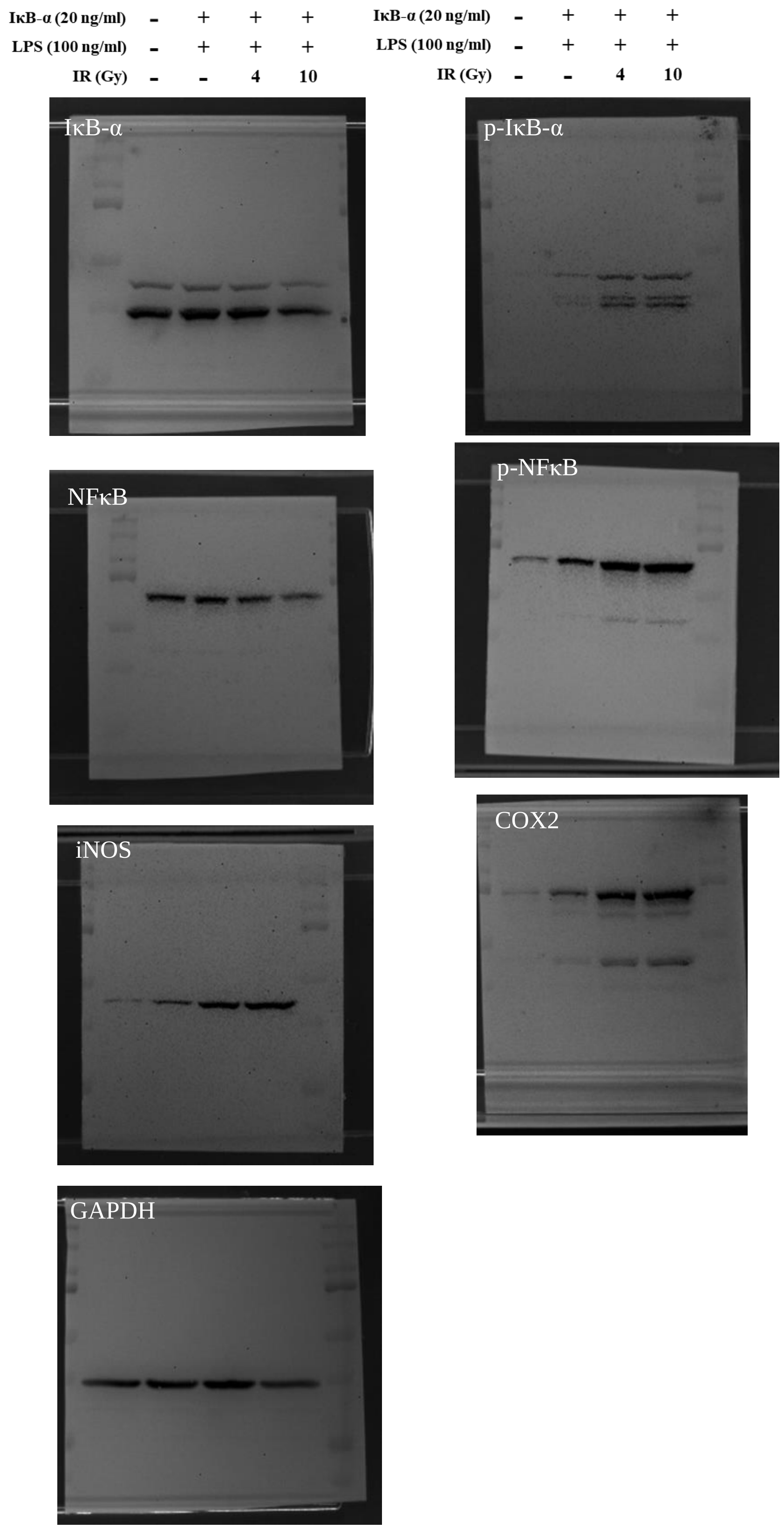

Supplement: S2 Raw images — (PDF) [file pone.0303434.s003.pdf]
